# Conditioning treatment with CD27 Ab enhances expansion and antitumor activity of adoptively transferred T cells in mice

**DOI:** 10.1007/s00262-021-02958-9

**Published:** 2021-05-24

**Authors:** Anna Wasiuk, Jeff Weidlick, Crystal Sisson, Jenifer Widger, Andrea Crocker, Laura Vitale, Henry C. Marsh, Tibor Keler, Li-Zhen He

**Affiliations:** 1 Celldex Therapeutics, Inc., 53 Frontage Road, Suite 220, Hampton, NJ 08827 United States; 2 Celldex Therapeutics, Inc., Celldex Therapeutics, Inc., 151 Martine Street, Fall River, MA 02723 USA

**Keywords:** Adoptive cell therapy (ACT), CD27 Ab, CD70-CD27 signaling, T cell depletion, Conditioning therapies

## Abstract

Cyclophosphamide plus fludarabine (C/F) are currently used to improve the expansion and effectiveness of adoptive cell therapy (ACT). However, these chemotherapeutics cause pan-leukopenia and adverse events, suggesting that safer and more effective conditioning treatments are needed to improve ACT outcomes. Previously, we reported that varlilumab, a CD27-targeting antibody, mediates *T*_reg_ -preferential T cell depletion, CD8-T cell dominant costimulation, and systemic immune activation in hCD27 transgenic mice and cancer patients. We reasoned that the activities induced by varlilumab may provide an effective conditioning regimen for ACT. Varlilumab pretreatment of h*CD27* ^+*/*+^m*CD27 *^*− /−*^ mice resulted in prominent proliferation of transferred T cells isolated from wild-type mice. These studies uncovered a critical role for CD27 signaling for the expansion of transferred T cells, as transfer of T cells from CD27 deficient mice or treatment with a CD70 blocking antibody greatly reduced their proliferation. In this model, varlilumab depletes endogenous h*CD27*^+*/*+^ T cells and blocks their subsequent access to CD70, allowing for more CD70 costimulation available to the m*CD27* ^+*/*+^ transferred T cells. CD27-targeted depletion led to a greater expansion of transferred T cells compared to C/F conditioning and resulted in longer median survival and more cures than C/F conditioning in the E.G7 tumor model receiving OT-I cell therapy. We propose that translation of this work could be achieved through engineering of T cells for ACT to abrogate varlilumab binding but preserve CD70 ligation. Thus, varlilumab could be an option to chemotherapy as a conditioning regimen for ACT.

**Supplementary Information:**

The online version contains supplementary material available at 10.1007/s00262-021-02958-9.

## Introduction

Adoptive cell therapy (ACT), including the transfer of genetically engineered T cells expressing a chimeric antigen receptor (CAR), or T cell receptor (TCR), to recognize tumor-associated antigens (TAAs), has emerged as breakthrough anti-cancer immunotherapy. In particular, CAR-T cell therapy has become an important approach in the management of hematological malignancies due to high rates of long-term remission [1–4]. For efficient in vivo expansion of transferred cells and therapeutic efficacy, lymphodepletion prior to ACT is required [5, 6]. Lymphodepletion removes a potential cytokine sink, i.e., reduces competition for the homeostatic cytokines IL-2, IL-7, and IL-15, eliminates regulatory T cells (*T*_reg_), and activates the innate immune system [5, 7, 8]. Currently, cyclophosphamide (C) alone or in combination with fludarabine (F) are commonly used as conditioning agents to induce lymphodepletion. However, the chemotherapeutics induce pan-leukopenia, which weakens innate immunity and diminishes humoral and cellular responses, including the responses to TAAs [9]. Furthermore, the *T*_reg_ reduction induced by C/F is not sustained and is even followed by a rebound [9–12]. Therefore, a safer and more effective conditioning treatment for T cell depletion is needed to improve the efficacy of ACT, especially to extend its efficacy into solid tumors.

CD27, unlike other members of the TNF receptor superfamily, is constitutively expressed on most T cells. CD27 costimulatory signaling is controlled by its ligand, CD70, which is transiently expressed upon activation of dendritic cells, B cells, and T cells, and is tightly regulated [13–15]. We have reported that CD27 is expressed at differential levels among T cell subtypes in the peripheral blood of cancer patients, and in the blood and lymphoid tissues of human CD27 transgenic mice (h*CD27*Tg), with the highest expression level on *T*_reg_ [16–18]. Targeting CD27 with the antibody (Ab) varlilumab (clone name 1F5, a human IgG1) and its mouse IgG2a isotype variant led to a *T*_reg_ -preferential T cell depletion in h*CD27*Tg mice, i.e., the extent of reduction among T cell subtypes was *T*_reg_  > CD4-T helper cells (CD4-Th) > CD8-T cells [16]. The same pattern of *T*_reg_-preferential T cell reduction following varlilumab administration was observed in the blood of cancer patients who participated in the phase I [17, 19] and phase II (manuscript in preparation) clinical trials. CD27-mediated T cell depletion resulted in increased ratios of CD8-T or CD4-Th cells to *T*_reg_, more CD8-T cells displaying the phenotype of central memory (CD127^+^CD44^+^CD62L^+^), and higher levels of proliferation (Ki-67^+^), activation (IFNγ^+^, TNFα^+^, IL-2^+^), and cytolysis (GzmB^+^) in lymphoid organs and tumor infiltrates [16]. In addition, varlilumab, through its agonistic activity, enhanced T cell responses in the context of MHC-TCR signal, upregulated cytokines and proinflammatory factors, and elevated the number and activation of antigen-presenting cells (APC) and NK cells in h*CD27*Tg mice [16, 18, 20] and in cancer patients [17]. Also, varlilumab is able to block hCD27 binding to both human and mouse CD70 [21]. All these activities of varlilumab, including its clinical benefit in some cancer patients and its well-established safety profile [17, 19], motivated us to investigate the potential of this Ab as a conditioning regimen for ACT. Here, we report that varlilumab pretreatment strongly promotes the expansion and persistence of adoptively transferred T cells and results in greater tumor killing capacity of ACT as compared to current conditioning agents in a solid tumor model.

## Materials and methods

### Mice

On the C57BL/6 background, h*CD27*Tg homozygous mice [18] express CD45.1 but not CD45.2. *CD27* knockout mice (m*CD27*KO) were generated using embryonic stem cell DNA homologous recombination, and the loss of gene expression was validated by flow cytometry (Fig. S1). h*CD27*^+*/*+^m*CD27*^*−/−*^ mice were derived by cross-mating h*CD27*Tg and m*CD27*KO mice. OT-I, *Rag2*^−/−^, and C57BL/6 wild-type (m*CD27*^WT^) mice were, respectively, purchased from Jackson Laboratory and Taconic Biosciences or bred in-house. All mice used in this study were housed under specific pathogen-free conditions in our animal facility and used in accordance with the guidelines established by the Institutional Animal Care and Use Committee (IACUC) at Celldex Therapeutics.

### Antibodies and reagents for in vivo applications

Development and characterization of varlilumab has been previously described [16, 18, 21]. Varli_mut_ is varlilumab on a mouse IgG1 backbone carrying a D265A mutation, which abrogates the interaction with all mouse FcγRs [16]. 2C2 is another clone of human IgG1 anti-human CD27 Ab isolated and characterized during the development of varlilumab. CD70 Ab (FR70) was purchased from Bio X cell. SIINFEKL peptide was synthesized by GenScript. C and F were purchased from Sigma-Aldrich and Toronto Research Chemicals, respectively. All Abs, isotype controls, and reagents used in vivo were endotoxin-free (< 1 EU/mg).

### Cell isolation, labeling, and infusion

CD3-, CD4-, or CD8-T cells were isolated from pooled spleen and peripheral lymph nodes (pLNs) of CD45.1^−^CD45.2^+^ m*CD27*^WT^, m*CD27*KO, or OT-I mice by negative selection using MACS isolation kits (Miltenyi Biotec). The isolated cells were labeled with fluorescent dye carboxyfluorescein succinimidyl ester (CFSE, Invitrogen) and transfused intravenously (i.v.) into h*CD27*^+/+^m*CD27*^−/−^ or *Rag2*^−/−^ mice. Recipient mice were pre-treated with 200 or 300 μg of varlilumab, hIgG1 isotype control, 2C2, or varli_mut_ by intraperitoneal (i.p.) injections. To block CD70-CD27 interactions, 200 μg of the CD70 Ab was injected i.p. on days -2, 1, 4, 7, and 10. C/F pretreatment was administered i.p. at 1 mg and 0.1 mg per dose, respectively. The doses of C and F used here were 2- to fivefold higher than that used in conditioning treatment for CAR-T cell therapy in ongoing clinical trials (NCT03549442, NCT03126864).

### Flow cytometry analysis

Spleen and pLNs were collected from recipient mice on days 7, 14, or 21 post T cell transfusion and flow cytometry analyses were performed to assess the depletion, proliferation, phenotype, and functional state of recipient and donor origin cells. CD45.1 and CD45.2 were used as congenic markers to distinguish between donor and recipient cells. Single cell suspensions were first stained with blue-fluorescent reactive dye (Invitrogen) to exclude dead cells from analysis, then for surface markers, and subsequently for intracellular or intranuclear molecules following fixation and permeabilization with the CytoFix/CytoPerm or Foxp3 Buffer Sets (BD Biosciences and eBioscience). Fluorescence dye-conjugated Abs for cell surface CD45.1 (A20), CD45.2 (104), CD3 (145-2C11), CD4 (GK1.5), CD8 (53–6.7), CD25 (7D4), CD19 (1D3), NK1.1 (PK136), CD11b (M1/70), and Foxp3 (MF23) were purchased from BD Biosciences, eBioscience, or BioLegend. *T*_reg_ were defined as CD4^+^Foxp3^+^ or CD4^+^CD25^+^ cells as indicated in figure legends, and CD4-Th cells were defined as CD4^+^Foxp3^−^. Data were acquired using a Canto II flow cytometer and analyzed using the FCS Express V4 software.

### Tumor challenge studies

E.G7 (ATCC), an EL4-derived ovalbumin-expressing thymoma, was grown in RPMI 1640/10% FBS/PenStrep/0.4 mg/ml G418, and 0.5 × 10^6^ cells were inoculated subcutaneously (s.c.) into the right flank of h*CD27*^+*/*+^m*CD27*^*−/−*^ mice. Varlilumab, or hIgG1, was injected i.p. 7 and 14 days after tumor inoculation. C/F were injected i.p. on days 13 and 14 post tumor inoculation. OT-I cells (2 × 10^6^) were transfused i.v. on day 16 followed by one dose of 20 μg of SIINFEKL peptide i.p. on day 17. Tumors were measured twice weekly and volumes were calculated using a modified ellipsoid formula [*V* = 1/2 (length x width^2^)]. Mice were euthanized upon reaching predefined endpoints approved by the IACUC.

### Identification of key amino acid (aa) residues on CD27 to distinguish binding of CD70 from varlilumab

Various truncated and mutated fragments of the CD27 extracellular domain, each of which were fused to a human kappa-chain and carried a flag-tag, were generated by recombinant protein techniques. Biotinylated CD70 and rhCD70-Fc were purchased from US Biologicals and Sino Biological, respectively. ELISA and biolayer interferometry (BLI, Fortebio Octet) were used to test varlilumab and CD70 binding to the various CD27 fragments.

### Statistical analysis

GraphPad Prism 8 software was used for statistical analysis. Data were expressed as mean and SD. Data shown in each graph are from a single study that are representative of 2–4 repeated studies, with 3–5 mice per group unless otherwise specified. Student’s *t* test, one-way and two-way ANOVA were used for comparison between two or multiple groups. Mantel–Cox test was used for survival curve comparison.

## Results

### Pretreatment with varlilumab enhances the expansion of adoptively transferred CD8-T cells

**Fig. 1:**
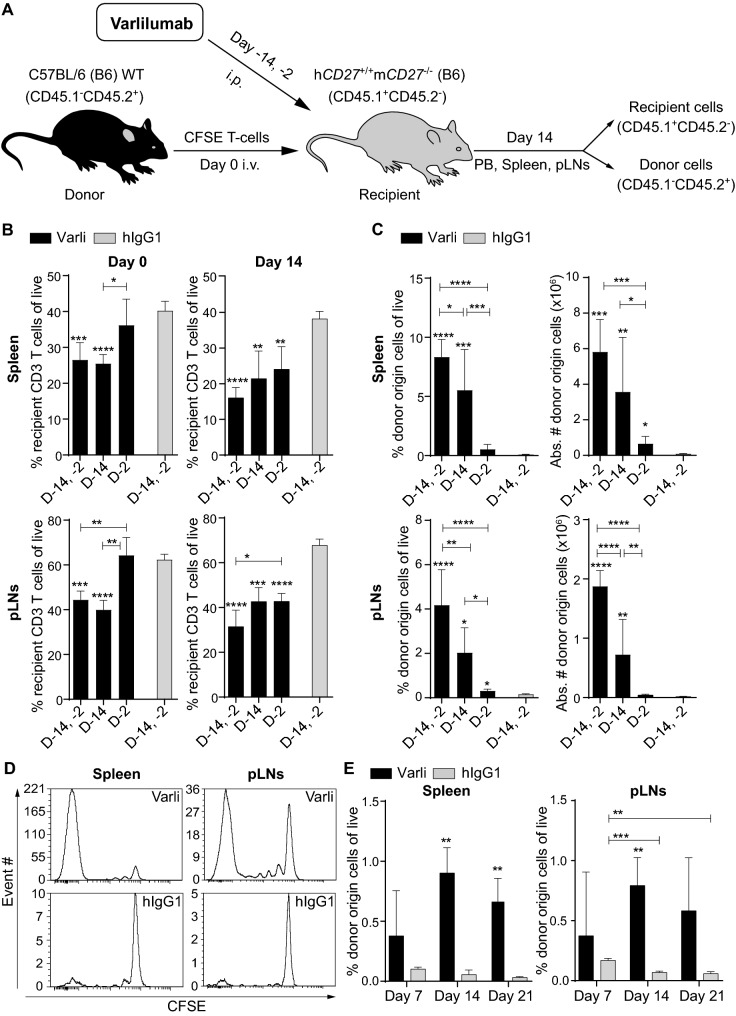
Optimal treatment with varlilumab leads to depletion of recipient T cells and expansion of donor CD8-T cells. **a** An adoptive T cell transfer schema illustrates the experimental design in this study. Additional modifications are specified in individual figure legends. **b, c** Two sets of h*CD27*^+/+^m*CD27*^−/−^ mice were injected with 300 μg of varlilumab or hIgG1 isotype control on days -14 or -2 or both. Spleens and pLNs were collected from one set of mice on day 0, without cell transfusion, and from the other set of mice 14 days post transfusion with 2 × 10^6^ CFSE-labeled WT CD8-T cells for the assessment of recipient cell depletion (B) and donor cell expansion (C). Percentages of recipient origin CD3-T cells (CD45.1^+^CD45.2^−^CD3^+^) and donor origin CD8-T cells (CD45.1^−^CD45.2^+^CD3^+^CD8^+^) out of total live cells in spleens and pLNs were determined by flow cytometry and the absolute numbers of donor origin cells were calculated based on total cell counts of spleens and pLNs. **d** Representative histogram plots of CFSE dilution assessed on day 14 show the proliferation of donor origin T cells in the spleen and pLNs of h*CD27*^+/+^m*CD27*^−/−^ mice treated as in (A). **e** h*CD27*^+/+^m*CD27*^−/−^ mice were treated with 200 μg of varlilumab, or hIgG1, on days -7 and -2 and transfused with 2 × 10^6^ CFSE-labeled WT CD8-T cells on day 0. Spleen and pLNs were analyzed on days 7, 14, and 21 post cell transfer. In all the graphs, the level of statistical significance is indicated as *p < 0.05, **p < 0.01, ***p < 0.001, ****p < 0.0001. Notations above bars indicate the statistical significance compared to hIgG1 isotype control; horizontal lines indicate statistical significance between the groups specified

We speculated that the T cell depleting activity of varlilumab would enhance the expansion of adoptively transferred T cells. Figure 1a is a schematic of the T cell transfer experiments. We first optimized varlilumab dosing and timing for recipient T cell depletion and donor cell expansion in h*CD27*^+/+^m*CD27*^−/−^ mice. Varlilumab administration on days -14 and -2 led to a significant decrease in CD3-T cells in spleen and pLNs at day 0, with further decrease by day 14, resulting in up to 50% reduction in T cells relative to control (Fig. 1b). Spleen and pLNs collected from h*CD27*^+/+^m*CD27*^−/−^ mice given the same regimen of varlilumab pretreatment and transfused with m*CD27*^WT^ CD8-T cells were subjected to the analysis of donor cell expansion. The greatest expansion of donor cells was achieved in mice pretreated with varlilumab on days -14 and -2, consistent with the depletion results (Fig. 1c). CFSE dilution validated that the increase in donor origin cells was indeed the consequence of their proliferation (Fig. 1d). Based on these results two doses of varlilumab, on days -14 or -7 and day -2, was selected for further studies.

To observe the peak and duration of the donor cell expansion, spleen and pLNs were collected from recipients at different timepoints post CD8-T cell transfusion. As shown in Fig. 1e, the donor origin cells were significantly increased on day 14 and sustained through day 21 in varlilumab-pretreated mice, compared to an overall decline in hIgG1-pretreated mice, indicating that varlilumab conditioning leads to long-lasting expansion of transferred T cells.

### Pretreatment with varlilumab favors the expansion of CD8-T cells over CD4-T cells

**Fig. 2:**
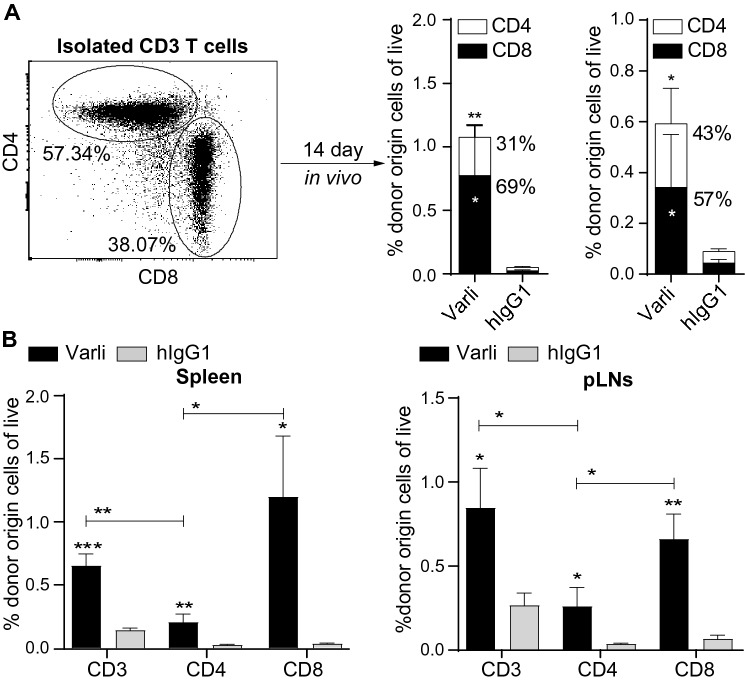
Varlilumab pretreatment favors the expansion of CD8-T cells over CD4-T cells. **a** Dot plot shows the proportion of CD4- and CD8-T cells within the isolated WT CD3-T cells. These CD3-T cells were labeled with CFSE and transfused at 2 × 10^6^ per mouse into h*CD27*^+/+^m*CD27*^−/−^ recipients pretreated with 300 μg of varlilumab, or hIgG1, on days -14 and -2. Stacked bar graphs show the percentages of donor origin cells out of total live cells in spleen and pLNs, with relative percentages of CD8- and CD4-T cells labeled, after 14 days’ in vivo expansion. **b** h*CD27*^+/+^m*CD27*^−/−^ mice were transfused with 3 × 10^6^ CFSE-labeled WT CD3-, CD4-, or CD8-T cells after pretreatment with 200 μg of varlilumab, or hIgG1, on days -14 and -2. Shown are percentages of donor origin cells out of total live cells in the spleen and pLNs after 14 days’ in vivo expansion. In all the graphs, the level of statistical significance is indicated as *p < 0.05, **p < 0.01, ***p < 0.001. Notations above bars indicate the statistical significance compared to hIgG1 isotype control; horizontal lines indicate statistical significance between the groups specified

We next compared the relative magnitude of expansion between CD8- and CD4-T cells in two studies. First, CD3-T cells were transfused into h*CD27*^+/+^m*CD27*^−/−^ mice after varlilumab pretreatment. While there were more CD4-T cells than CD8-T cells in the isolated CD3-T cells before transfer, the ratio was reversed after 14 days’ in vivo expansion (Fig. 2a). Second, equal number of CD3-, CD4-, or CD8-T cells were transfused into h*CD27*^+/+^m*CD27*^−/−^ mice after varlilumab pretreatment. All three populations of T cells expanded more in recipients pretreated with varlilumab compared to isotype control, but the number of donor origin CD4-T cells at day 14 was significantly smaller than that of donor origin CD8- or CD3-T cells (Fig. 2b). Together, the data indicate that varlilumab conditioning favors the expansion of CD8-T cells over CD4-T cells.

### CD27 signaling is critical for the expansion of adoptively transferred CD8-T cells

**Fig. 3:**
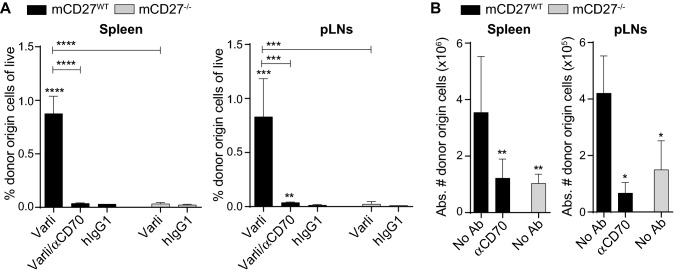
Expansion of transferred CD8-T cells is abrogated or reduced upon loss of CD27 signaling. **a** h*CD27*^+/+^m*CD27*^−/−^ mice were injected with 200 μg of varlilumab, or hIgG1, on days -14 and -2 with or without 200 μg CD70 blocking Ab on days -2, 1, 4, 7, and 10. CFSE-labeled CD8-T cells (3 × 10^6^) isolated from m*CD27*^WT^ or m*CD27*^−/−^ mice were transfused on day 0 and assessed for expansion 14 days later. Shown are the percentages of donor origin cells out of total live cells in spleen and pLNs. **b** Immunodeficient *Rag2*^−/−^ mice were left untreated or given 200 μg of CD70 blocking Ab on days -2, 1, 4, 7, and 10. On day 0 mice were transfused with CFSE-labeled CD8-T cells (2 × 10^6^) isolated from m*CD27*^WT^ or m*CD27*^−/−^ mice. Spleens and pLNs were harvested 14 days later and absolute numbers of donor origin cells are shown. In all the graphs, the level of statistical significance is indicated as *p < 0.05, **p < 0.01, ***p < 0.001, ****p < 0.0001. Notations above bars indicate the statistical significance compared to hIgG1 isotype control in (A) or to *Rag2*^−/−^ mice transfused with m*CD27*^WT^ CD8-T cells without CD70 Ab in (B); horizontal lines indicate statistical significance between the groups specified

To understand if CD27 signaling in donor cells plays a role on their expansion, we compared the proliferation of CD8-T cells in varlilumab-pretreated h*CD27*^+/+^m*CD27*^−/−^ mice in the presence or absence of CD27 signaling. As shown in Fig. 3a, the elimination of CD27 signaling in donor cells, either by injecting a CD70 Ab that blocks CD70-CD27 interaction or by transfusing CD8-T cells isolated from m*CD27*^*−/−*^ mice, abolished the enhancement of donor cell expansion following varlilumab pretreatment. CD27 signaling in donor cells is ascribed to the interaction with CD70 expressed on recipient cells. This interaction is not blocked by the presence of varlilumab because it does not cross-react with mCD27 expressed on donor cells. CD70 was not detected on the expanded donor cells, or recipient CD8-T cells (Fig. S2).

Further, we tested the effect of CD27 signaling on donor cell expansion in untreated *Rag2*^−/−^ mice that completely lack T and B cells. Donor cells isolated from m*CD27*^WT^ mice expanded to a significantly greater extent than m*CD27*^−/−^ donor cells did, or m*CD27*^WT^ donor cells did in the presence of CD70 blocking Ab (Fig. 3b). These data corroborate that CD27 signaling potentiates the expansion of transferred cells.

### Selectively blocking recipient T cells from accessing CD70 by varlilumab contributes to the enhanced expansion of adoptively transferred CD8-T cells

**Fig. 4:**
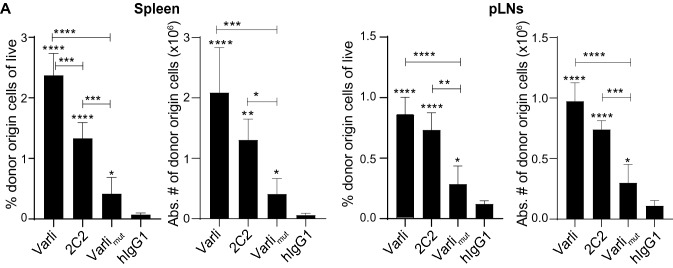
Competition for CD70 from recipient endogenous cells diminishes the proliferation of transferred CD8-T cells following varlilumab pretreatment. h*CD27*^+/+^m*CD27*^−/−^ mice were injected with 300 μg of the CD27 Abs varlilumab (depleting, agonistic, and blocking), 2C2 (depleting, agonistic but not blocking) or varli_mut_ (not depleting, nor agonistic but blocking), or isotype control hIgG1 on days -14 and -2 and transfused with 2 × 10^6^ CFSE-labeled WT CD8-T cells on day 0. Shown are the percentages and absolute numbers of donor origin cells out of total live cells in the spleen and pLNs after 14 days of in vivo expansion. In all the graphs, the level of statistical significance is indicated as *p < 0.05, **p < 0.01, ***p < 0.001, ****p < 0.0001. Notations above bars indicate the statistical significance compared to hIgG1 isotype control; horizontal lines indicate statistical significance between the groups specified

To further understand the mechanisms of varlilumab in enhancing expansion of transferred cells, we dissected its depleting and agonistic activities from its blocking activity by using the anti-CD27 Ab 2C2, which shows T cell depletion and agonism similar to varlilumab but does not block CD70 binding, or the Fc-mutated varli_mut_ that only retains ligand blocking activity but lacks depletion and agonism activities (Fig. S3), for pretreatment. 2C2, like varlilumab and varli_mut_, does not bind mCD27 (data not shown), and thus these three Abs exert direct effects only on the h*CD27*^+/+^m*CD27*^−/−^ recipient cells and not on the m*CD27*^WT^ donor cells. As shown in Fig. 4, either depletion without blocking CD70-CD27 signaling (2C2) or blocking without depletion (varli_mut_) increased the expansion of donor cells relative to isotype control. The expansions in these two groups, however, were not as great as that in mice pretreated with varlilumab, indicating that blocking ligand binding to CD27 on recipient’s T cells, in addition to T cell depletion, plays a role in the enhancement of donor cell expansion.

### Varlilumab pretreatment leads to better conditioning effects versus C/F regimens

**Fig. 5:**
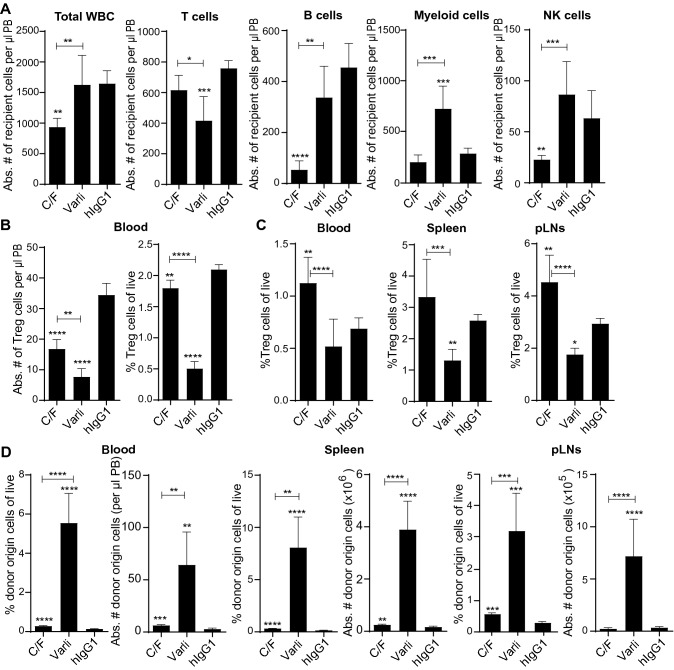
Comparison of varlilumab pretreatment versus C/F conditioning regimen in recipient cell depletion and donor cell expansion. **a** h*CD27*^+/+^m*CD27*^−/−^ mice were treated with 300 μg of varlilumab, or hIgG1, on days -14 and -2, or C/F (1 and 0.1 mg, respectively) on days -4, -3, -2. Mice were bled on day 0 prior to WT CD8-T cell transfer. Whole blood was assessed for cell depletion by flow cytometry analysis. Shown are absolute numbers of indicated cell population per μl blood. Cell populations are defined as follows: CD8-T cells = CD3^+^CD8^+^, CD4-T cells = CD3^+^CD4^+^CD25^−^, B cells = CD19^+^, Myeloid cells = CD19^−^CD4^−^CD8^−^NK1.1^−^CD11b^+^, and NK cells = CD19^−^CD4^−^CD8^−^NK1.1^+^. **b** The percentage and absolute numbers of *T*_reg_ cells (CD3^+^CD4^+^CD25^+^) were assessed from the same mice as in (A). **c** and **d** Blood, spleen, and pLNs were collected on day 14 from mice pretreated as in (A) and transfused with 2 × 10^6^ CFSE-labeled WT CD8-T cells on day 0. Shown in (C) are percentages of recipient origin *T*_reg_ cells, which were defined as CD45.1^+^CD45.2^−^CD3^+^CD4^+^CD25^+^ in the blood and CD45.1^+^CD45.2^−^CD4^+^Foxp3^+^ in the spleen and pLNs. Shown in (D) are the percentages and absolute numbers of donor origin cells in the 3 tissues indicated. In all the graphs, the level of statistical significance is indicated as *p < 0.05, **p < 0.01, ***p < 0.001, ****p < 0.0001. Notations above bars indicate the statistical significance compared to hIgG1 isotype control; horizontal lines indicate statistical significance between the groups specified

Chemotherapy with C/F is the current standard conditioning regimen for ACT. We compared recipient cell depletion and donor CD8-T cell expansion upon pretreatment with varlilumab or C/F as a conditioning treatment. The profiles of cell populations analyzed on day 0 in the blood of h*CD27*^+/+^m*CD27*^−/−^ mice (the day that T cells would be transferred), were as expected, showing that varlilumab significantly reduced the T cell compartment accompanying with increased myeloid cells and no significant impact on NK cells, which are hCD27^neg/low^. The T cell compartment decreases are driven by depletion of *T*_reg_, and CD4-T cells with more modest decreases in CD8-T cells [16], while γδT cells and NKT cells are also modestly decreased (data not shown). In contrast, C/F reduced total white blood cell counts, especially B and NK cells, when analyzed on day 0, the day that T cells would be transferred (Fig. 5a). Although both regimens preferentially depleted *T*_reg_ relative to other subtypes of T cells, varlilumab reduced the numbers of *T*_reg_ to a greater extent than C/F did (Fig. 5b). Moreover, as shown in Fig. 5c, the number of *T*_reg_ remained low in varlilumab-treated mice while they recovered, or even rebounded, to higher than control levels in C/F-treated mice by day 14, evidencing that *T*_reg_ depletion was maintained longer in varlilumab-treated mice than in C/F-treated mice.

Next, h*CD27*^+/+^m*CD27*^−/−^ mice given varlilumab, C/F, or isotype control were transfused with m*CD27*^WT^ CD8-T cells to measure donor cell expansion at day 14. Strikingly, the percentage and absolute numbers of donor origin cells in blood, spleen, and pLNs were significantly higher in varlilumab-pretreated mice than that in C/F-pretreated mice, although a significantly increased expansion of donor T cells was also detected with C/F-pretreatment, relative to the control group (Fig. 5d).

### Varlilumab conditioning leads to stronger antitumor activity of adoptively transferred TCR-T cells compared to C/F regimens

We used OT-I cells, which were isolated from ovalbumin peptide SIINFEKL-specific TCR transgenic mice, and ovalbumin-expressing E.G7 tumor as a model system to test conditioning treatment with varlilumab versus C/F in the enhancement of antitumor activity of ACT. We have previously documented that E.G7 tumor-bearing mice responded to varlilumab administration (30–60% cure rate) when treatment was initiated 5 days (or earlier) after tumor inoculation [16, 18, 21], but not when treatment is further delayed. Similar results were observed with 3 mg of C given once or divided into 3 weekly doses initiated within 7–8 days post E.G7 tumor inoculation (Fig. S4). In the present studies, which aimed to show the effectivness of varlilumab when used as a conditioning regimen, mice were treated with varlilumab (day 7, 14) and C/F (day 13, 14) when minimal direct antitumor efficacy is observed (Fig. S5), thus allowing for the evaluation of their conditioning effects on the antitumor potency of the transferred OT-I cells.

**Fig. 6:**
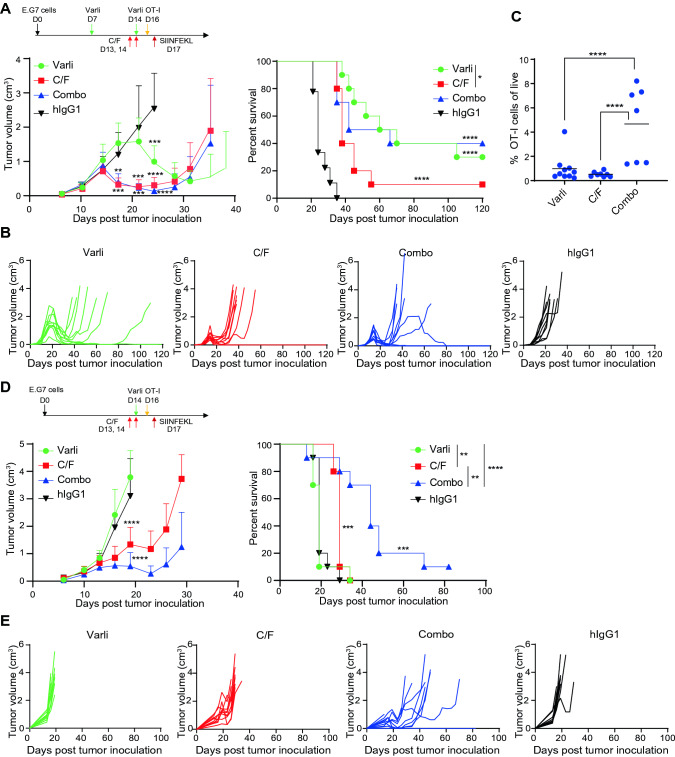
Comparison of the conditioning effects of varlilumab versus C/F on the enhancement of antitumor activity of OT-I cells. **a** Groups of 10 h*CD27*^+/+^m*CD27*^−/−^ mice were inoculated s.c. with 0.5 × 10^6^ E.G7 cells on day 0. Tumor-bearing mice were treated with 300 μg of varlilumab, or hIgG1, on days 7 and 14, or C/F (1 and 0.1 mg, respectively) on days 13 and 14, or the combination of both (Combo), and transfused with 2 × 10^6^ OT-I cells on day 16 followed by 20 μg of SIINFEKL peptide in saline the next day, as shown in the schema. Tumors were measured twice weekly, and mice were monitored daily for survival. Shown are tumor volumes (mean ± SD), and a Kaplan–Meier survival plot. The tumor growth curve of each group was ended when the first mouse reached endpoint and euthanized, while the survival was followed up by 120 days post tumor inoculation. **b** Data from (A) presented as tumor growth curves in individual mice. **c** Whole blood was collected at day 34 from the mice in (A) and assessed for the presence of circulating OT-I cells in the three surviving groups. **d** Groups of 10 h*CD27*^+/+^m*CD27*^−/−^ mice received the same tumor inoculation and all the treatments as in (A) except for varlilumab on day 7 only, as shown in the schema. Shown are tumor volumes (mean ± SD and in individual mouse) and a Kaplan–Meier survival plot. The tumor growth curve of each group was ended when the first mouse reached endpoint and euthanized, while the survival was followed up over 80 days post tumor inoculation. **e** Data from (D) presented as tumor growth curves in individual mice. In all the graphs, the level of statistical significance is indicated as *p < 0.05, **p < 0.01, ***p < 0.001, ****p < 0.0001. Notations above survival curves indicate the statistical significance compared to hIgG1 isotype control; vertical lines indicate statistical significance between the groups specified

As shown in the mean and individual tumor growth curves in Fig. 6a and b, on the day of OT-I cell transfer (16 days post tumor inoculation), the average tumor volume had reached ~1000 mm^3^ in the groups of control and varlilumab-pretreated mice, whereas the average tumor volume was significantly smaller in the C/F- and combo-pretreated groups, indicating that the E.G7 tumor partially responded to the chemotherapy but not to varlilumab. Compared to hIgG1 control, both conditioning regimens enhanced antitumor efficacy of the transfused OT-I cells. However, varlilumab pretreatment resulted in longer median survival and a higher cure rate than C/F conditioning did, despite the delay in tumor regression in varlilumab-pretreated group (Fig. 6a). Therefore, the therapeutic benefit observed here is dependent on varlilumab-induced expansion of OT-I cells, which is observed particularly in the combination group by blood sampling taken at day 34 (Fig. 6c). Mice that were treated with combo of C/F and varlilumab had a comparable survival to mice conditioned with varlilumab alone, despite the direct killing effect exerted by C/F as shown by the identical tumor growth curves between C/F and combo groups at the early stage. Delaying the varlilumab treatment to day 14 (2 days prior to OT-1 transfer) did not enhance the antitumor activity, consistent with the weak donor cell expansion in mice pretreated with varlilumab on day -2 (Fig. 1c), yet potentiated C/F conditioning effects (Fig. 6d and e). These data emphasize the stronger and longer lasting conditioning effects derived from two doses of varlilumab, without additive benefit by adding C/F, to the optimal effectiveness of varlilumab conditioning (Fig. 6a).

### Potential approach to adapt varlilumab conditioning into ACT clinical practice for patients

**Fig. 7:**
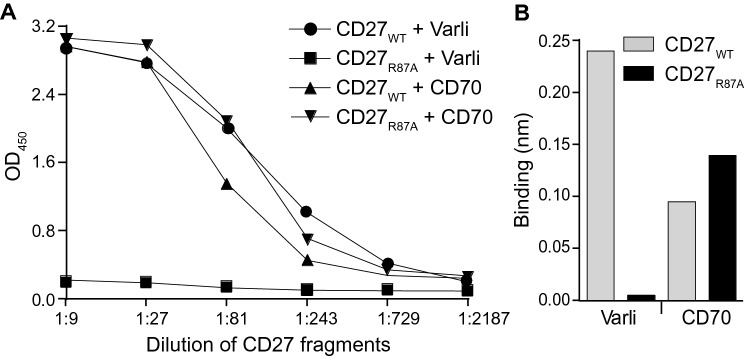
CD27_R87A_ mutation abrogates the binding of varlilumab, but not CD70, to the CD27 receptor. **a** ELISA. CD27_WT_-flag or CD27_R87A_-flag fragment-containing supernatants were serially diluted and captured by microplate-bound anti-flag Ab. Wells were then incubated with varlilumab, or CD70-biotin, and binding was detected with HRP-conjugated secondary anti-human IgG Ab, or streptavidin, followed by substrate. Shown are readouts of OD_450_ of varlilumab or CD70 binding against dilutions of indicated CD27 fragments. **b** BLI. A Fortebio Octet analysis was performed using anti-human Fc biosensors loaded with either varlilumab, recombinant human CD70-Fc, or buffer alone, and then exposed to CD27 fragment-containing supernatants. Binding was reported as nanometer (nm) shifts at the end of the association step after subtraction of non-specific background

To translate our findings into human clinical practice, it is critical to protect the adoptively transferred T cells from depletion by the preinjected varlilumab that remains in circulation of the patient, while retaining CD70-mediated costimulation. This could potentially be achieved by engineering T cells to express a mutated form of CD27 that allows CD70, but not varlilumab, binding. ELISA and BLI assays using multiple truncated CD27 fragments determined that both CD70 and varlilumab bound to an N-terminal fragment spanning aa residues 1–110 of the mature CD27 polypeptide. We then selectively replaced aa in the human CD27 sequence within this range with the mouse counterpart where the sequences diverged between the two species since varlilumab does not bind mouse CD27. A single point mutation of residue 87 (CD27_R87A_) completely abrogated varlilumab, but not CD70, binding as demonstrated by ELISA and BLI assays (Fig. 7). Therefore, it can be reasonably speculated that the introduction of the R87A substitution into endogenous CD27 during T cell preparation for ACT would allow costimulation by endogenous CD70 but prevent depletion by varlilumab.

## Discussion

In this study, we demonstrate that a conditioning treatment using the CD27 Ab, varlilumab, leads to remarkable and selective expansion of transferred CD8-T cells and uncovers the critical importance of CD27 signaling in this expansion. The data support two mechanisms involved in the enhancement of transferred T cell expansion by varlilumab. First, as with conventional conditioning treatments [6, 7, 22–24], varlilumab-mediated reduction in lymphocyte numbers provided more space for and prevented competition for available homeostatic cytokines by the transferred T cells. Whereas, conditioning with a non-depleting varlilumab variant significantly impaired the expansion of transferred cells. Second, varlilumab selectively blocked CD70 access to the hCD27-expressing recipient T cells and thus skewed CD70-mediated costimulation toward the mCD27 expressing transferred cells. The importance of this mechanism is reinforced by demonstrating that a CD27 Ab that has equivalent depleting activity, but no CD70-CD27 blocking activity, was less effective at inducing donor T cell expansion. Additionally, varlilumab’s agonistic activity, including the transient upregulation of multiple cytokines, growth factors, proinflammation factors, and the resulting elevation in myeloid cells, including APC, [17, 19, 20] might also play a role in the enhancement of transferred T cell expansion.

We demonstrate that the optimal expansion of transferred CD8-T cells upon varlilumab conditioning treatment is dependent on the costimulatory CD70-CD27 signal specifically in the transferred T cells that express mCD27 but not hCD27. The importance of this interaction was borne out by showing that T cells from m*CD27*^−/−^ mice did not expand when transferred into varlilumab-pretreated recipients and, similarly, that T cell expansion could be abrogated by injecting CD70 blocking Ab into the recipients. Importantly, the role of CD27 signaling on the expansion of transferred cells was also demonstrated in T cell deficient *Rag2*^*−/−*^ recipients, where m*CD27*^WT^ donor cells expanded more efficiently in the absence of CD70 blocking Ab versus in the presence of CD70 blocking Ab, or when using m*CD27*^*−/−*^ donor cells. Thus, the importance of CD27 signaling in the expansion of adoptively transferred cells is generally applicable and not restricted to varlilumab conditioning. We believe the source of CD70 is most likely host APCs as we did not observe CD70 expression on the proliferating transferred T cells (Figure S2); however, we cannot rule out the possibility of contribution from cell-autonomous mCD70-mCD27 signaling on transferred T cells. It is yet to be determined if CAR- or TCR-incorporated costimulatory signals, such as CD28 and 41BB, can compensate for the requirement of, or synergize with, endogenous CD27 signal in cells for adoptive transfer.

CD27 signaling triggered with CD70 or varlilumab has dominant costimulatory effects on CD8-T cells, as compared to CD4-T cells, and is able to trigger CD4-independent CD8-T cell activation [16, 25–27]. These observations are consistent with studies showing intrinsic differences in the way that homeostatic survival signals are transmitted in CD4- and CD8-T cells, with CD4-T cells having lower capacity for survival and slower division rates compared to CD8-T cells upon transfer into syngeneic lymphopenic hosts. [28, 29].

In the mouse models described, varlilumab was found to be superior to the standard C/F conditioning regimen in *T*_reg_-preferential T cell depletion, the magnitude of expansion of transferred T cells, and the antitumor activity. The chemotherapy regimen induced broad depletion in all leukocytes, in agreement with other studies that showed a lack of TAA priming [9, 10]. Varlilumab-induced depletion was primarily limited to T cells and was accompanied by elevated numbers and activation status of APC [16, 20]. Furthermore, while *T*_reg_ recovered rapidly and rebounded in C/F conditioned mice, in line with others’ observations [9–12], *T*_reg_ numbers remained low in varlilumab-pretreated recipients 14 days post cell transfusion, likely due to the longer pharmacokinetics and pharmacodynamics of varlilumab, similar to that observed in monkey study and cancer patients [17, 21]. The advantages of sustained *T*_reg_ depletion and the presence of APC, together with direct and indirect effects of CD27 costimulatory signaling, allows the conditioning treatment with varlilumab to potentiate the transferred T cells and enable the responses of recipient’s endogenous T cells to TAAs derived from a growing tumor or vaccination [24, 30, 31], thus extending antitumor immunity beyond the single target recognized by CAR-T cells or TCR-T cells.

Identification of the CD27_R87A_ substitution, which preserves CD70 binding but completely abrogates varlilumab binding, makes the findings of this mouse study translatable into human clinical practice. We propose that this aa substitution can be introduced by gene editing when T cells are being ex vivo engineered to express the CAR or TCR. The CRISPR-Cas9 approach has been demonstrated feasible in the knock-in or knock-out of genes such as *TCR* and *PDCD-1*, or to correct a pathogenic *IL2RA* mutation in human primary T cells and is being pursued in clinical trials [32–34]. As such, T cells carrying the mutated CD27 will be analogous to T cells from m*CD27*^WT^ mice used in the present study in their ability to receive costimulatory signals from CD70 without the concern of depletion by residual varlilumab, as illustrated in Fig. S6.

Taken together, the present study reports a critical role for the CD27/CD70 signaling pathway in ACT, and demonstrates that varlilumab pretreatment offers a novel, potentially safer, and more effective approach as a conditioning regimen for ACT in a mouse model system and has the potential to be translated into clinical practice. The profound *T*_reg_ depletion, prominent CD8-T cell expansion, and enhanced opportunity for antigen spreading following varlilumab conditioning may improve ACT efficacy in solid tumors [24, 35]. The robust in vivo expansion of transferred T cells may reduce the number of cells required for transfusion, thus providing an opportunity for ACT in patients from whom adequate or qualified T cells are difficult to collect due to lymphopenia from primary disease or prior therapies. In addition, varlilumab pretreatment may also be a better option for ACT in indications other than cancer, such as HIV and HBV [36, 37], where chemotherapy as preconditioning is less acceptable.

## Supplementary Information

Below is the link to the electronic supplementary material.

Supplementary file1 (_2020 2438 kb)

## Data Availability

All data generated or analyzed during this study are included in this manuscript.

## Abbreviations

Ab: Antibody
ACT: Adoptive cell therapy
APC: Antigen-presenting cells
*C*: Cyclophosphamide
CAR: Chimeric antigen receptor
CD4-Th: CD4-T helper cells
CFSE: Carboxyfluorescein succinimidyl ester
*F*: Fludarabine
FcγRs: Fc-gamma receptors
h*CD27*Tg (h*CD27*^+^^/^^+^): Human *CD27* transgenic mice
i.p.: Intraperitoneally
i.v.: Intravenously
m*CD27*KO (m*CD27*^−^^/^^−^): *CD27* Knockout mice
m*CD27*^WT^: C57BL/6 wild-type mice
pLNs: Peripheral lymph nodes
s.c.: Subcutaneously
TAAs: Tumor-associated antigens
TCR: T cell receptor
*T*_reg_: Regulatory T cells
