# Supplementary material for: Conditioning treatment with CD27 Ab enhances expansion and antitumor activity of adoptively transferred T cells in mice
Source: Cancer Immunol Immunother. 2021 May 24;71(1):97–109. doi: 10.1007/s00262-021-02958-9 (PMC8739312; doi:10.1007/s00262-021-02958-9)
Supplement: Supplementary file 1 — Supplementary file1 (_2020 2438 kb) [file 262_2021_2958_MOESM1_ESM._2020]

Figure S1

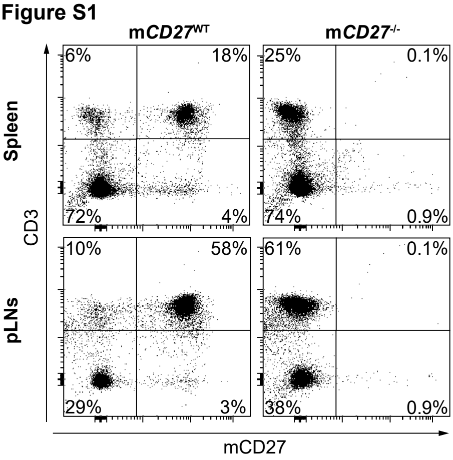

Figure S1. Lack of mCD27 expression in T cells from m*CD27*$^{-/-}$ mice was confirmed by FACS. Spleen and pLNs from naive m*CD27*^{WT} and m*CD27*$^{-/-}$ mice were collected and processed into a single cell suspension. Cells were then stained for T cell markers and mCD27 (clone 034, Sinobiological). Representative dot plots show the expression of mCD27 in WT but not m*CD27*$^{-/-}$ mice. Data are representative of one experiment with n = 5 mice/group.

Figure S2

A

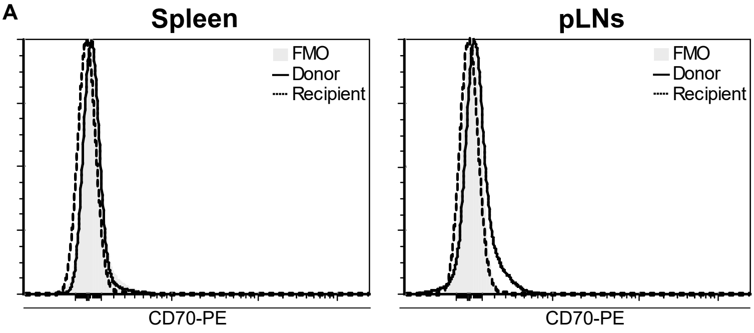

Figure S2. No CD70 expression was detected on CD8 T cells after varlilumab pretreatment and cell transfer. h*CD27*$^{+/+}$m*CD27*$^{-/-}$ mice were injected with 300 μg of varlilumab or hIgG1 on days -14 and -2 and were transfused with WT CD8-T cells on day 0. On day 14 spleen and pLNs were collected and processed into a single cell suspenion and cells were stained for CD70 expression (clone FR70). Histograms show representative expresson of CD70 on expanded donor origin cells and recipient endogenous CD8 T cells.

Figure S3

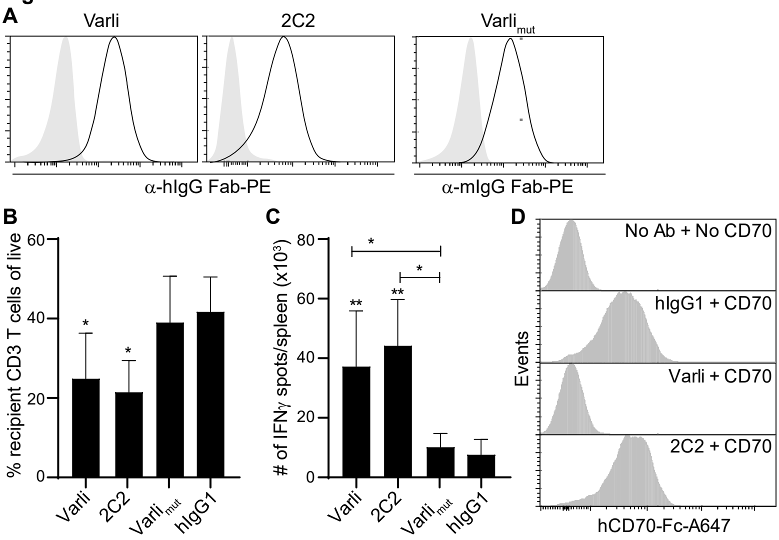

Figure S3. 2C2 has comparable depleting and agonistic activities with varlilumab but no blocking activity, while varli$_{mut}$ has no depleting or agonistic activities.
(**A**) CCRF-CEM leukemia cells were incubated for 30 minutes on ice, in the presence of Fc block, with varlilumab, 2C2, varli$_{mut}$, hIgG1, or mIgG1, and Ab binding to CD27 was detected with a secondary goat anti-human, or anti-mouse, IgG-Fab Ab, as indicated. Solid lines show CD27 Ab binding and gray filled histograms are the isotype controls. Equal binding to CD27 by all three Abs is shown. (**B**) h*CD27*$^{+/+}$m*CD27*$^{-/-}$ mice were injected with 300 μg of varlilumab, varli$_{mut}$, 2C2, or hIgG1 on days -14 and -2. Shown are percentages of CD3 T cells in the blood on day 0 (n = 5 mice/group). (**C**) h*CD27*Tg mice were injected with 5 mg OVA and 50 μg of varlilumab, varli$_{mut}$, 2C2, or hIgG1 on day 0. Spleens were collected 7 days later for ELISPOT analysis. Shown are SIINFEKL-specific IFNγ spot numbers per spleen (n = 5 mice/group). (**D**) Splenocytes from h*CD27*$^{+/+}$m*CD27*$^{-/-}$ mice were incubated with 100 μg Ab or hIgG1, as indicated, for 30 minutes on ice and then stained with a fluorescently labeled human CD70-Fc fusion protein (0.5 μg) and Abs for cell surface markers. Shown is a histogram of hCD70-Fc staining on CD4$^+$CD25$^+$ T$_{reg}$. While hCD70 binding was the same on cells preincubated with 2C2 (2C2 + CD70) or hIgG1 isotype control (hIgG1 + CD70), there was no hCD70 binding detected on cells preincubated with varlilumab (Varli + CD70). Data are representative of 2 independent studies; *p< 0.05, **p<0.01 and indicate statistical significance compared to isotype control or between groups as specified by the horizontal line.

Figure S4

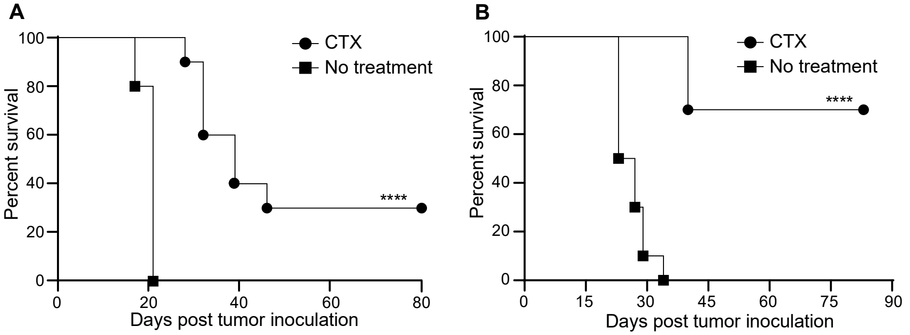

Figure S4. Cyclophosphamide significantly extends survival of E.G7 bearing h*CD27*Tg mice. (**A**) E.G7 cells (1 x 10^6) were inoculated s.c. on day 0 into hCD27Tg mice (N=10) and treated with C (1 mg) i.p. weekly for 3 weeks. Treatment was initiated on day 7 when average tumor volume reached ~50 mm^3. (**B**) E.G7 cells (1 x 10^6) were inoculated s.c. on day 0 and treated with C (3 mg) i.p. once on day 8 when average tumor volume reached ~50 mm^3. Groups of 10 mice/group were used in both experiments and ****p<0.0001 indicate the statistical signifiance compared to untreated controls.

Figure S5

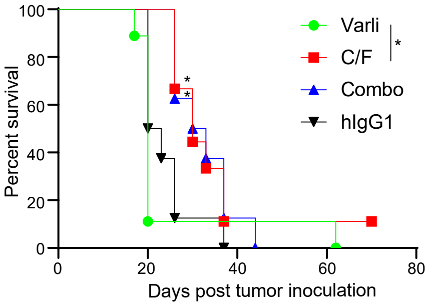

Figure S5. No antitumor activity results from delayed dosing of varlilumab in h*CD27*Tg mice without OT-I cell transfer. E.G7 cells (0.5 x 10^6) were inoculated s.c. on day 0 into h*CD27*Tg mice (N=10). On days 7 and 14 varlilumab (300 μg) was administered i.p., and on days 13 and 14 mice were treated i.p. with C (1 mg) and F (100 μg). The combo group received both varlilumab and C/F. C/F slightly extend overal survival, the combination of C/F and Varli had no additive effect compared to C/F, and varlilumab alone was ineffective.

Figure S6

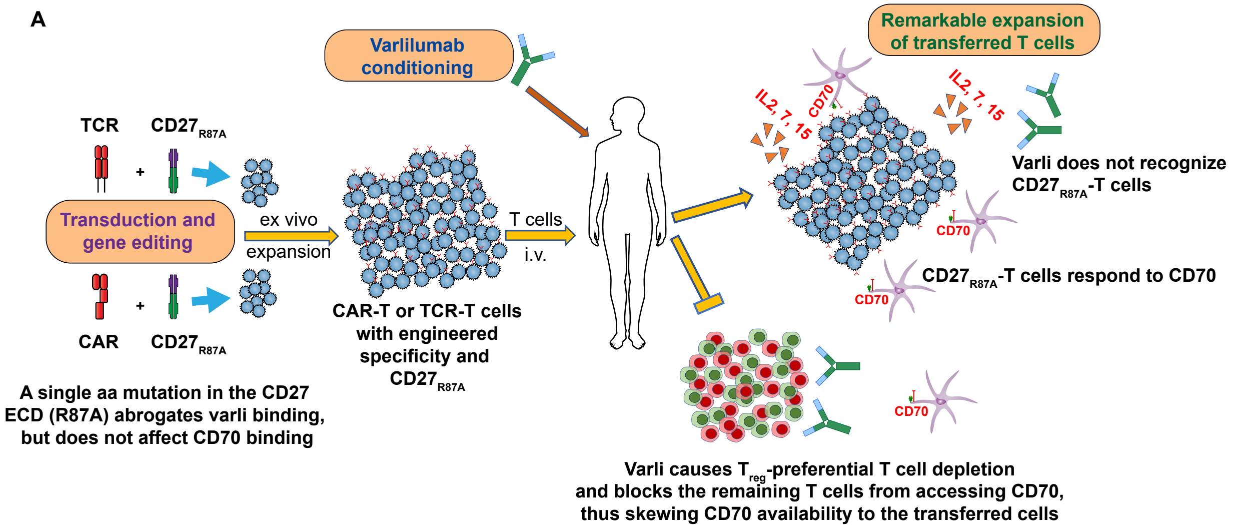

Figure S6. Hypothetical working model of varlilumab conditioning for ACT in clinical practice. Patients are pretreated with varlilumab leading to T_{reg}-preferential T cell depletion and reduced endogenous T cell proliferation due to its blocking of CD70 engagement. A CD27 R87A substitution is introduced into T cells during the preparation of CAR-T, or TCR-T, cells to avoid depletion after transfer by preinjected varlilumab. Upon adoptive transfer the $CD27_{R87A}$-engineered CAR-T, or TCR-T, cells encounter increased availability of common cytokines and CD70 costimulation, concurrent with decreased T_{reg} suppression, resulting in their robust expansion and activation in vivo.
